# Urinary Normetanephrine for the Diagnosis of Canine Pheochromocytoma via Liquid Chromatography–Mass Spectrometry

**DOI:** 10.3390/vetsci13020159

**Published:** 2026-02-06

**Authors:** Matteo Petini, Andrea Zoia, Tommaso Furlanello, Luca Magna, Riccardo Masti, Jose Sanchez del Pulgar, Francesca Maria Bertolini

**Affiliations:** 1 San Marco Veterinary Clinic and Laboratory, Via dell’Industria 3, 35030 Veggiano, Italy; matteo.petini@sanmarcovet.it (M.P.); andrea.zoia@sanmarcovet.it (A.Z.); luca.magna@sanmarcovet.it (L.M.); francesca.bertolini@sanmarcovet.it (F.M.B.); 2 CREA Research Centre for Food and Nutrition, Via Ardeatina 546, 00178 Rome, Italy; jose.sanchezdelpulgar@crea.gov.it

**Keywords:** pheochromocytoma, catecholamines, LC–MS/MS, urinary biomarkers, veterinary endocrinology, reference intervals

## Abstract

Pheochromocytomas (PHEO) are rare adrenal medullary tumours characterised by catecholamine secretion. Accurate diagnosis relies on the sensitive measurement of metanephrine (MN) and normetanephrine (NMN); however, the application of liquid chromatography–mass spectrometry (LC–MS/MS) in veterinary medicine remains limited. This study aimed to develop and analytically validate an LC–MS/MS method for the simultaneous quantification of urinary MN and NMN in dogs, establish reference intervals (RIs) for MN- and NMN-to-creatinine ratios (uMN:CR and uNMN:CR), and preliminarily assess the diagnostic utility of uNMN:CR for PHEO detection. The RIs were 2.2–78.9 nmol/mmol for uMN:CR and 4.4–77.4 nmol/mmol for uNMN:CR. Dogs with PHEO showed significantly higher uNMN:CR values compared with dogs with other adrenal masses, non-adrenal disease, or healthy controls. Using the upper RI limit as a threshold, sensitivity and specificity were 91.7% and 35.7%, respectively. Receiver operating characteristic analysis identified an optimal uNMN:CR cut-off of 203.7 nmol/mmol, yielding 78.6% sensitivity, 83.3% specificity, and an overall diagnostic accuracy of 85% (95% CI: 0.68–1.00). These findings support LC–MS/MS as a reliable approach for urinary metanephrine analysis and uNMN:CR as a valuable biochemical tool for PHEO identification in dogs.

## Simple Summary

Pheochromocytomas are rare adrenal tumours in dogs producing catecholamines that derive from the chromaffin cells and often cause non-specific clinical signs, making diagnosis challenging. This study describes the development and validation of a non-invasive urine test using liquid chromatography–tandem mass spectrometry to measure hormone metabolites. Reference ranges were established in healthy dogs, and affected dogs showed higher values than dogs with other conditions. This analytical approach provides accurate and stable results and supports veterinarians in the biochemical identification of pheochromocytoma, improving diagnostic confidence and clinical decision-making.

## 1. Introduction

Pheochromocytomas (PHEOs) are rare yet clinically significant neuroendocrine tumours originating from the chromaffin cells of the adrenal medulla. These tumours release excessive catecholamines, including epinephrine and norepinephrine, leading to various clinical signs that are often nonspecific but may become life-threatening. Reported manifestations include irregular heart rate, elevated blood pressure, lethargy, increased respiratory rate, abdominal discomfort, and heightened thirst and urination. The diagnosis of PHEO is challenging due to the overlap of these symptoms with other endocrine or systemic diseases and the need for highly sensitive and specific biochemical assays [1,2].

Diagnostic investigation combines clinical evaluation, imaging, and laboratory testing, with particular focus on the measurement of catecholamines and their metabolites, specifically metanephrine (MN) and normetanephrine (NMN). Unlike the episodic release of catecholamines, metanephrines are produced continuously by PHEO, providing more stable and sensitive diagnostic markers. Their quantification in plasma or urine has therefore become a cornerstone in PHEO diagnostics in both human and veterinary medicine [1,3].

In human medicine, multiple analytical techniques have been developed for the determination of catecholamines and their metabolites, including high-performance liquid chromatography with electrochemical detection (HPLC-ECD), gas chromatography–mass spectrometry (GC-MS), and, more recently, liquid chromatography–tandem mass spectrometry (LC–MS/MS) [4,5]. The latter is now considered the reference method due to its superior sensitivity, specificity, and analytical reliability, enabling a more accurate distinction between affected patients and healthy individuals [4,5]. In veterinary medicine, several studies have demonstrated that urinary catecholamine quantification provides a non-invasive approach for the biochemical diagnosis of pheochromocytoma (PHEO) [6,7,8].

Despite the increasing clinical adoption of LC–MS/MS for its recognition as the gold standard for accurate quantification [9,10,11], the veterinary literature on catecholamine quantification using this technique remains extremely limited [6,7,8,12,13,14,15,16]. A recent study by van den Berg et al. [17], provided reference intervals (RI) for plasma, urinary, and salivary free metanephrines in healthy dogs and also quantified metanephrines in dogs with PHEO, hypercortisolism (HC), or non-adrenal illness (NAI). However, a key challenge remains in determining whether plasma or urine samples offer more reliable results in canine diagnostics, as discrepancies persist in the literature regarding the optimal sample type for metanephrine analysis [1]. Even though the triggers for catecholamine secretion in dogs are usually unknown [1], urinary NMN is an attractive diagnostic test. It is also the only measurement that appears largely unaffected by stress related to veterinary handling and sample collection, further supporting its use in routine clinical practice [2]. In advancing the application of this laboratory method, it is essential to account for possible species-specific physiological and metabolic differences. These differences require rigorous validation of the analytical procedures, with particular attention to pre-analytical variables such as plasma and urine collection techniques, sample preservation, and creatinine normalisation. Nonetheless, to the authors’ knowledge, no previous studies have reported the application of a fully validated LC–MS/MS method for the quantification of urinary catecholamines in dogs. In particular, the literature lacks comprehensive studies addressing both analytical validation, including method performance parameters such as accuracy, precision, sensitivity, and stability, and clinical validation through the establishment of RI and diagnostic cut-off values in well-characterised animal cohorts.

In light of these considerations, the first aim of this study was to develop and validate a robust, accurate, and rapid LC–MS/MS method for the simultaneous quantification of NMN and MN in canine urine. The second aim was to establish RIs for these analytes in healthy dogs. Finally, the third aim was to preliminarily assess the diagnostic performance of urinary NMN, measured using this validated method, for the diagnosis of PHEO.

## 2. Materials and Methods

### 2.1. Study Population

Client-owned dogs presented at the San Marco Veterinary Clinic (Veggiano, Italy) between October 2022 and October 2025 were retrospectively included.

#### 2.1.1. Healthy Group

Dogs of various breeds, ages, and body weight presented for routine wellness checks and/or undergoing routine procedures (e.g., preoperative evaluations for neutering or dental care, oestrous cycle monitoring, or general health screening) were included in the healthy group. Health status was confirmed based on a complete clinical evaluation, including medical history review, physical examination, complete blood count, serum biochemistry, and urinalysis.

#### 2.1.2. Other Groups

Dogs with documented adrenal masses by diagnostic imaging and with a cytological and/or histological diagnosis of pheochromocytoma were included in the PHEO group. Dogs with documented adrenal masses on imaging, in which pheochromocytoma was excluded by cytology and/or histology, were included in the non-PHEO group. Critically ill dogs with various diseases, but without adrenal masses, were also enrolled for comparison in the NAI group.

Dogs were not eligible if they were receiving any medication capable of influencing catecholamine concentrations (e.g., metoclopramide, alpha- and beta-blockers, calcium-channel blockers, or glucocorticoids).

### 2.2. Urine Samples

Urine samples were collected either by spontaneous voiding or cystocentesis in all dogs. Urine MN and NMN were measured in the PHEO group and non-PHEO group as part of their diagnostic work-up within 24 h of urine collection. In the healthy and NAI groups, MN and NMN were measured from residual urine samples obtained for urinalysis. Samples were either analysed fresh (i.e., within 24 h of collection) or immediately aliquoted into polypropylene tubes and stored frozen (−20 °C) for no longer than two months. All samples were thawed only once, immediately before analysis.

### 2.3. Reagents and Materials

LC–MS grade methanol and formic acid were obtained from Merck (LiChrosolv^®^, Darmstadt, Germany). Analytical grade ammonium acetate and ammonium formate were also purchased from Merck. Certified reference standards of MN hydrochloride and NMN hydrochloride (>99% purity) and their deuterated analogues (metanephrine-d3 and normetanephrine-d3) were procured from commercial suppliers (Merck, Darmstadt, Germany). Ultrapure water was produced using a Milli-Q system (Millipore, Bedford, MA, USA). Stock and working solutions of native and deuterated standards were prepared fresh according to validated protocols and stored at −20 °C until use.

### 2.4. LC–MS/MS Instrumentation and Chromatographic Conditions

Quantitative analysis was performed using an Agilent 1260 Infinity HPLC system coupled to an Agilent 6490 Triple Quadrupole mass spectrometer (Agilent Technologies, Santa Clara, CA, USA) equipped with an electrospray ionisation source in positive ion mode. Chromatographic separation was achieved on an Agilent Pursuit PFP column (150 × 2.0 mm, 3 µm particle size; Agilent Technologies) maintained at 40 °C. The mobile phases consisted of 10 mM ammonium formate in water with 0.1% formic acid (A) and methanol (B), delivered at a flow rate of 0.3 mL/min. The gradient programme was set as follows: 0–1.0 min, 0% B; 1.0–4.0 min, 0–60% B; 4.0–6.0 min, 60% B; and 6.1 min, return to 0% B, with a total run time of 8 min. The MS source parameters were: drying gas temperature 200 °C, drying gas flow 16 L/min, sheath gas temperature 250 °C, sheath gas flow 12 L/min, nebulizer pressure 30 psi, capillary voltage 3500 V, nozzle voltage 0 V, iFunnel high-pressure RF 150 V, and low-pressure RF 60 V. Metanephrine and NMN were monitored in multiple reaction monitoring mode with the following transitions: MN (*m*/*z* 180.1 → 165.1, 148.0), NMN (*m*/*z* 166.1 → 137.2, 123.1, 109.2), metanephrine-d3 (*m*/*z* 183.1 → 168.0, 151.1, 123.0), and normetanephrine-d3 (*m*/*z* 169.1 → 134.0, 106.1). Collision energies (CE) and cell acceleration voltages (CAV) were optimised for each transition. Data acquisition and quantitative analysis were performed using Agilent MassHunter software (version 7.0).

### 2.5. Sample Preparation

Aliquots of urine (50 µL) were pipetted into 1.5 mL polypropylene tubes and spiked with 20 µL of deuterated internal standard mixture (final concentration 1 µg/mL). After the addition of 450 µL of 10 mM ammonium acetate buffer, samples were vortex-mixed for 5 min. Solid-phase extraction was performed using Strata X-CW weak cation exchange cartridges (30 mg/mL; Phenomenex, Torrance, CA, USA). Cartridges were conditioned with 1 mL of methanol followed by 1 mL of ammonium acetate buffer, and the entire sample volume was loaded. After washing with 1 mL of ammonium acetate buffer and 1 mL of methanol, analytes were eluted with 2 × 500 µL of 5% ammonium hydroxide in methanol. A total of 200 µL of the eluate was evaporated to dryness under vacuum at 60 °C for 20 min and reconstituted in 200 µL of water containing 0.1% formic acid.

### 2.6. Determination of Urinary Creatinine

Urinary creatinine (uCr) levels were quantified with the Atellica^®^ CH Analyzer (Siemens Healthcare Diagnostics Inc., Duisburg, Germany) using the Creatinine_2 (Crea_2) test. This method applies to a kinetic adaptation of the Jaffé reaction, in which creatinine interacts with alkaline picrate to yield a coloured complex. The rate of change in absorbance reflects the concentration of creatinine, while built-in correction factors help minimise interference from non-specific chromogens. The assay’s dynamic range for urine specimens extends from 3.00 to 245.00 mg/dL, with 3.00 mg/dL serving as the quantification threshold. All procedures adhered to the manufacturer’s instructions, and internal quality control measures were consistently applied.

### 2.7. Analytical Method Validation

Method validation was conducted in accordance with the European Medicines Agency (EMA) guidelines for bioanalytical method validation [18]. The parameters assessed included linearity, lower limit of quantification (LLOQ), intra- and inter-assay precision and accuracy, recovery, matrix effect, carry-over, and stability. Calibration curves were constructed from nine calibration points ranging from 1 to 500 ng/mL using weighted (1/x^2^) regression. The LLOQ was set at 1 ng/mL, meeting the acceptance criteria of accuracy and precision within ±20%. Precision and accuracy were evaluated at three QC levels (low, medium, and high), with acceptance limits of ±15%. Recovery was calculated by comparing analyte responses in extracted urine samples versus neat standards, and matrix effects were assessed by comparing post-extraction spiked urine with solvent-based standards. Carryover was evaluated by injecting blank samples after the highest calibrator, while stability testing included freeze–thaw cycles, bench-top exposure, and long-term storage at −20 °C.

### 2.8. Stability

Stability testing was performed on a quality control sample at a single concentration level, analysed in triplicate, to evaluate the robustness and reliability of the analytical method under various storage and handling conditions. The stability analyses were conducted using a single freshly collected urine sample, which was immediately aliquoted into the number of portions required for the planned tests and stored under the investigated conditions. The stability of the samples was assessed over 7 days (days 1, 3, and 7) for room temperature and refrigerated (4 °C) storage. Stability for frozen samples (−20 °C) was evaluated for both MN and NMN also at 180 and 365 days. In addition to these storage conditions, thermal stress stability was assessed for both MN and NMN by exposing the samples to an elevated temperature of 40 °C for the full 7-day period, with analyses performed at 1, 2, 3, and 7 days. This comprehensive approach ensured that the method’s performance was thoroughly tested across both typical and extreme storage conditions, simulating real-world scenarios such as shipping or temporary exposure to non-ideal environments. The stability of urinary creatinine under similar storage conditions and time frames was not assessed, as it has been previously demonstrated [19,20].

### 2.9. Reference Interval

Reference intervals for the urinary metanephrine-to-creatinine ratio (uMN:CR) and urinary normetanephrine-to-creatinine ratio (uNMN:CR) were established using 41 residual urine samples obtained from clinically healthy, client-owned dogs. uMN:CR and uNMN:CR values were calculated by dividing urinary MN and NMN concentrations, expressed in nanomoles per millilitre (nmol/mL), and by urinary creatinine concentrations, expressed in millimoles per millilitre (mmol/mL). The resulting ratios were reported as nmol/mmol.

### 2.10. Statistical Analysis

Statistical analysis was conducted using Reference Value Advisor (V2.1) for Microsoft Excel (2016). Continuous data were tested for normality of distribution with the Shapiro–Wilk test and summarised by mean and standard deviation (SD) or median and interquartile range ([IQR], 25th and 75th percentiles) and range (minimum and maximum values), depending on their distribution and sample size. Outliers were detected using the Dixon–Reed test. Non-parametric methods with bootstrap resampling were applied to determine reference intervals, as recommended by the IFCC–CLSI C28-A3 guidelines for sample sizes below 120. The Kruskal–Wallis test was utilised to compare the values of uNMN:CR among groups (Pheo, HC, NAI, healthy), with post hoc pair-wise comparisons performed using the Mann–Whitney U test with Bonferroni correction. Finally, receiver operator characteristic (ROC) curve analysis was performed to identify the optimal cut-off value of uNMN:CR for distinguishing dogs with PHEO from those with other adrenal masses. For all statistical tests, the significance level was set at α = 0.05.

## 3. Results

### 3.1. Study Population

Forty-one dogs were included in the healthy group. Of these, 9 were mongrels and 32 were purebred dogs, including Border Collies (*n* = 3), Labrador Retrievers (*n* = 2), Lagotto Romagnolo (*n* = 2), and one each of Akita Inu, Australian Cattle Dog, Australian Shepherd, Basset Hound, Beagle, Cavalier King Charles Spaniel, Chihuahua, Chow Chow, Czechoslovakian Wolfdog, Dachshund, German Shorthaired Pointer, Great Dane, Italian Greyhound, Jack Russell Terrier, Lessinia and Lagorai Shepherd, Pekingese, Romanian Carpathian Shepherd Dog, Rough Collie, Saarloos Wolfdog, Shetland Sheepdog, Shiba Inu, Siberian Husky, Toy Poodle, West Highland White Terrier, and Standard Poodle. There were 25 females (19 spayed and 6 intact) and 16 males (10 intact and 6 neutered). The median age was 48 months (IQR, 22.5–96 months), and the median body weight was 18.1 kg (IQR, 9.15–24.7 kg). The most common reason for analysis was clinical examination for a general health check (*n* = 28), followed by odontological procedures (9) and pre-neutering evaluations (4). Regarding sampling methods, cystocentesis was predominantly used, while free catch samples were taken in a smaller number of cases.

The PHEO group comprised 12 dogs, of which 7 were mongrels and 5 were purebred, including one each of French Bulldog, Italian Hound, Toy Poodle, Yorkshire Terrier, and Welsh Terrier. The median age was 150 months (IQR, 119–182.5 months). In four dogs, the diagnosis of pheochromocytoma was confirmed by histopathology, whereas in the remaining eight dogs, it was supported by cytological findings.

The non-PHEO group included 14 dogs, of which 3 were mixed-breed and 11 were purebred, including two Jack Russell Terriers and one each of Bolognese, Border Collie, Chihuahua, Dachshund, Spanish Greyhound, Labrador Retriever, Lagotto Romagnolo, Shih Tzu, and Yorkshire Terrier. There were nine female dogs (all spayed) and five male dogs (three intact and two neutered). The median age was 126.5 months (IQR, 108–152.5 months). Nine dogs were diagnosed with adrenal cortical tumours based on histopathology (cortical carcinoma, *n* = 7; cortical adenoma, *n* = 2), while in the remaining five dogs, cytological findings were highly suggestive of adrenal cortical disease (cortical neoplasia, *n* = 4; cortical hyperplasia, *n* = 1). Among these 14 dogs, a diagnosis of adrenal-dependent hyperadrenocorticism was supported by laboratory testing in seven dogs. In six dogs, the hormonal secretory activity of the adrenal lesion could not be clearly determined, and in one dog, hyperaldosteronism was suspected based on increased serum aldosterone concentration.

Lastly, the NAI group included 16 dogs, of which 5 were mixed-breed, and 11 were purebred, including two Jack Russell Terriers, two Labrador Retrievers, and one each of American Staffordshire Terrier, Cane Corso, Italian Greyhound, Maremmano–Abruzzese Sheepdog, and Schnauzer. The median age was 88 months (IQR, 35.2–122.3 months). The final diagnoses in this group included immune-mediated disease (*n* = 4), bite-related trauma (3), (non-adrenal) neoplasia (3), infectious disease (2), renal disease (1), cardiac disease (1), hepatic disease (1), and endocrine disease (1).

### 3.2. Method Validation

The calibration curves for urinary MN and NMN showed excellent linearity over the range of 1–500 ng/mL, with a coefficient of determination (R^2^) consistently exceeding 0.99. Back-calculated concentrations for all calibration points were within ±15% of their nominal values, confirming the accuracy of the calibration curves. The LLOQ was established at 1 ng/mL, with signal-to-noise ratios greater than 10 and accuracy and precision within the ±20% tolerance as per EMA guidelines. The limit of detection (LOD) was determined to be approximately 0.5 ng/mL, with a signal-to-noise ratio greater than 3:1, confirming the sensitivity of the assay (Table 1 and Table 2).

Precision and accuracy were rigorously evaluated across five quality control (QC) levels: LLOQ, low, medium and high concentrations, and upper limit of quantification (ULOQ). Results at each level, including the LLOQ, consistently showed interassay accuracy ranging from 97.2% to 107% for NMN and from 96.8% to 100.8% for MN, with coefficients of variation (CV%) remaining below 15%. This demonstrated excellent reproducibility and reliability of the method across the validated concentration range (Table 1 and Table 2). The matrix effect (ME%) values were close to 100%, indicating negligible ion suppression or enhancement from the canine urine matrix (Table 1 and Table 2). Carryover was negligible for both MN and NMN. Following injection of the highest calibration standard (500 ng/mL), analyte responses observed in subsequent blank samples were below 20% of the LLOQ, while internal standard responses were below 5%.

### 3.3. Stability

Throughout all times and thermal conditions tested, analyte concentrations remained within predefined acceptance criteria, confirming the method’s robustness and reliability under varying environmental conditions (Table 3).

### 3.4. Reference Interval and Diagnostic Performance

The median uMN:CR in healthy dogs was 26.1 nmol/mmol (IQR, 16.9–37). The sample size was sufficient to establish a RI of 2.2–78.9 nmol/mmol by the non-parametric method (90% CI for lower limit, 1.8–11.9; 90% CI for upper limit, 75.3–79.0).

For uNMN:CR, the median in healthy dogs was 38.2 nmol/mmol (IQR, 22.1–53.9), and the calculated RI was 4.4–77.4 nmol/mmol (90% CI for lower limit, 3.9–19.6; 90% CI for upper limit, 70.1–77.5).

**Figure 1:**
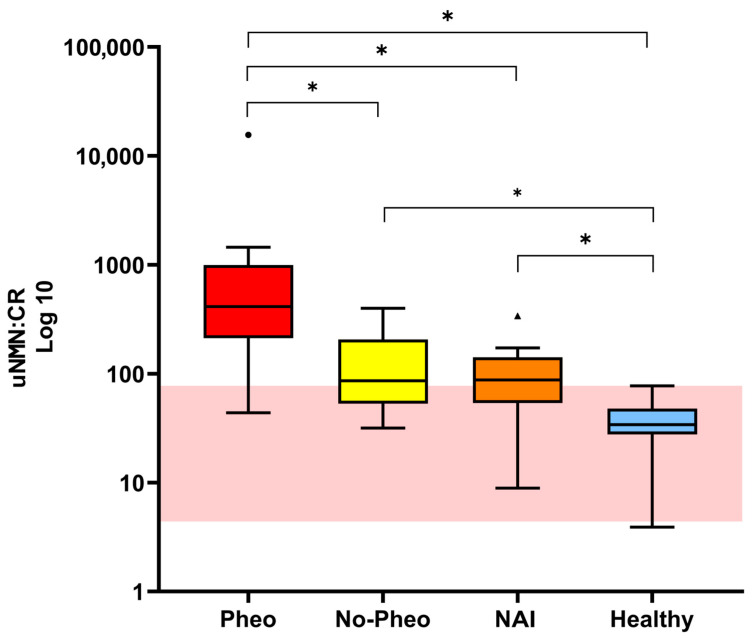
Box-and-whisker plots for urinary normetanephrine-to-urinary creatinine ratio (uNMN:CR) in hyperkalemic dogs with pheochromocytoma (Pheo, *n* = 12), dogs with adrenal masses other than pheochromocytoma (no-Pheo, *n* = 14), NAI (*n* = 16), and healthy dogs (*n* = 41). The bounds of the boxes are the 1st (the lower) and 3rd (the upper) quartiles; the black line across the boxes represents the median (2nd quartile). The whiskers correspond to the data still within the 1.5 interquartile. The circle and the triangle are outlier values (more than 1.5 interquartile range away from the closest end of the box). The shaded region represents the reference interval for uNMN:CR. The *Y*-axis values are plotted on a log10 scale. *, statistical significance between the groups.

The uNMN:CR values for each group are reported in Table 4. Statistical analysis showed that the uNMN:CR was significantly higher in dogs with PHEO compared to the other three groups (Table 4 and Figure 1). Also, the uNMN:CR was significantly higher in both NAI dogs and dogs with adrenal masses other than PHEO compared to healthy dogs (Table 4 and Figure 1). Using the upper RI of the uNMN:CR, the overall sensitivity and specificity of the test in identifying PHEO dogs was 91.7% and 35.7%, respectively. The positive predictive value and negative predictive value were 55% and 83.3%, respectively.

**Figure 2:**
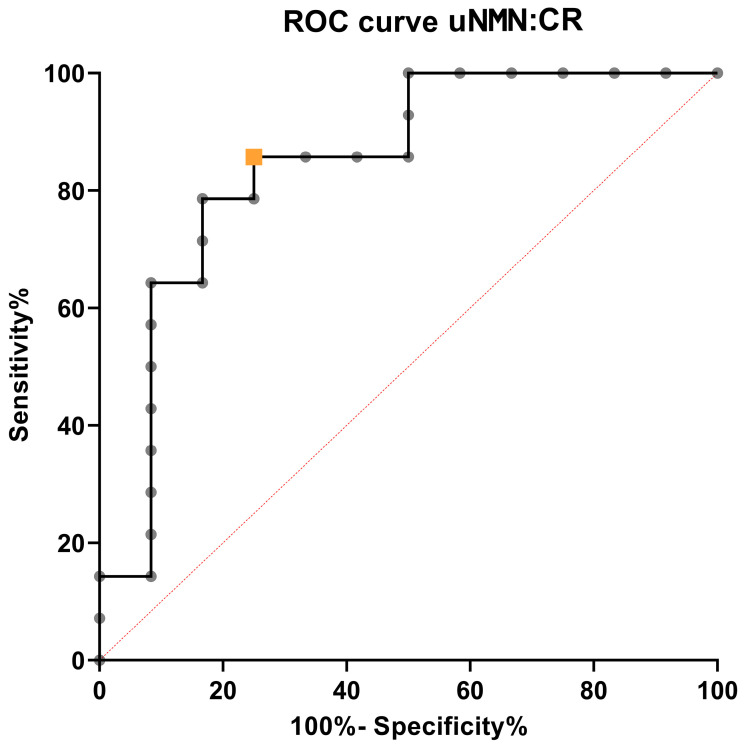
Receiver operating characteristic curves of the urinary normetanephrine-to-urinary creatinine (uNMN:CR) to discriminate dogs with pheochromocytoma from dogs with adrenal masses of a different type. The orange square is the Youden Index.

Finally, the optimal uNMN:CR cut-off value identified by the ROC curve analysis (i.e., the value that maximised the sum of sensitivity and specificity [Youden index]) in discriminating PHEO from non-PHEO dogs was 203.7 nmol/mmol. At this value, the cut-off sensitivity was 79% (95% CI, 52–92) and the specificity was 83% (95% CI, 55–97). The diagnostic accuracy of uNMN:CR in discriminating dogs with PHEO from non-PHEO dogs was 85% (95% CI, 68–100), (Figure 2). Using this cut-off value, 12 dogs with PHEO were correctly identified. The two dogs with PHEO with uNMN:CR below this value had the following concentrations: 43.8 nmol/mmol and 160.8 nmol/mmol.

## 4. Discussion

The present study pursued two primary objectives: (1) the development and analytical validation of an LC–MS/MS method for the quantification of uMN and uNMN in dogs, and (2) the assessment of the diagnostic performance of the uNMN:CR for the identification of dogs with PHEO. Together, these objectives integrate methodological validation with clinical application, providing a more robust framework for the biochemical investigation of adrenal medullary disease in veterinary medicine.

Although LC–MS/MS is universally regarded as the gold-standard analytical approach and is routinely employed in human medicine for the quantification of catecholamine metabolites [21,22,23], the available literature is largely focused on human experimental and clinical applications [24]. Its use in veterinary diagnostics remains limited due to restricted instrument availability and fewer laboratories with mass-spectrometry expertise, resulting in lack of species-specific analytical validation studies. Within this context, the present study demonstrates that the proposed LC–MS/MS method enables accurate, precise, and reproducible quantification of NMN and MN in canine urine. A key analytical contribution of this study concerns the comprehensive stability assessment, which is largely absent from the veterinary literature. Both uNMN and uMN, as well as their respective urinary creatinine ratios, demonstrated stability for up to 7 days across multiple storage conditions, including room temperature, refrigeration, freezing, and thermal stress at 40 °C. When stored at −20 °C, sample stability was maintained for at least one year. This high degree of stability supports flexible sample handling, delayed analysis, long-term storage, and retrospective testing, all of which are essential for routine diagnostic workflows, without compromising analytical reliability. A similar finding has been reported in human urine, where uMN and uNMN measured by LC–MS/MS remained stable for up to 29 weeks of frozen storage, further confirming the robustness of these analytes under prolonged preservation conditions [25]. The stability of these metabolites under a range of pre-analytical conditions and during long-term storage facilitates routine clinical implementation, batch analysis, multicenter studies, and interlaboratory transport.

The established LLOQ and ULOQ enabled reliable detection of both basal and pathologically elevated concentrations. Normalisation of uMN and uNMN to urinary creatinine reduces dilution-related effects, a major contributor to biological variability in canine urine [8,25,26]. Measurement of the uNMN:CR in spot urine samples has also been proposed in human medicine as a practical alternative to the more laborious 24 h urine collection [26,27].

Compared with immunoassays, enzymatic assays, or chromatographic methods requiring derivatization, this LC–MS/MS protocol provides superior selectivity, minimises cross-reactivity, and reduces procedural variability [11,27], thereby offering a robust and analytically reliable approach for the biochemical evaluation of catecholamine metabolites.

These analytical achievements form the foundation for the clinical results reported in the subsequent sections of the study, where the clinical application for the uNMN:CR alone in the diagnosis of canine PHEO was explored, considering that several studies previously showed its superiority over the uMN:CR [6,7,8,28]. Consistent with those earlier findings, our results confirmed that uNMN:CR is significantly higher in dogs with PHEO compared to dogs with different adrenal masses, dogs with non-adrenal diseases, and healthy dogs. According to the ROC curve analysis, this test shows a good diagnostic accuracy in discriminating dogs with PHEO from dogs with other adrenal disorders. However, some overlap in uNMN:CR values between PHEO and non-PHEO groups was observed, in line with previous reports [7,8], resulting in good specificity and moderate sensitivity when the test was applied at the Youden-index-derived cut-off value. This result is similar to a recent study in which the specificity was slightly lower [3]. Such a discrepancy may be attributable to differences in study populations. Specifically, in the study by Waldron et al. [3], the number of dogs with adrenal adenomas or carcinomas in the non-PHEO group diagnosed with adrenal dependent hyperadrenocorticism was not reported, whereas in our study, 7 of the 14 dogs classified as non-PHEO had adrenal dependent hyperadrenocorticism. This is important because hyperadrenocorticism is a differential diagnosis for PHEO, not only because of overlapping clinical signs and diagnostic imaging features [29], but also because endogenous glucocorticoids may increase the catecholamine production [30]. Additional complexity arises because endocrine neoplasms such as glucocorticoid-producing adrenocortical tumours or adrenocorticotropic hormone (ACTH)-secreting pituitary tumours frequently coexist with PHEO, making its diagnosis challenging. Moreover, clinical signs, laboratory abnormalities, and imaging findings also may substantially overlap among these disorders, hindering the clear differentiation of PHEO from other concurrent endocrinopathies [2]. For these reasons, when uNMN:CR values are elevated, clinicians should first rule out hyperadrenocorticism as a differential diagnosis and, ultimately, confirm the biochemical suspicion of PHEO (supported by high uNMN:CR values) through cytological and/or histopathological evaluation of the adrenal mass.

Values of uNMN:CR below the cut-off of 203.7 nmol/mmol, but above the reference interval (RI), do not completely exclude a diagnosis of PHEO. Similarly, values within the RI cannot definitively rule out the disease. In the present study, one dog in the PHEO group had a uNMN:CR concentration within the RI, and its uMN:CR (53.5 nmol/mmol) was also within the reference range. This finding supports the existence of silent pheochromocytomas, in which catecholamine secretion is absent, hormone concentrations remain within normal limits, or catecholamine synthesis is completely lacking [31]. Alternatively, uNMN:CR may exhibit insufficient sensitivity in certain cases. In humans, plasma NMN serves as a more reliable biomarker, though its performance hinges on multiple pre-analytical, analytical, and post-analytical variables [9,32]

Despite the substantial analytical and clinical advantages offered by the present method, several limitations of this study should be acknowledged. First, the stability test was performed on a single quality control sample, which allowed assessment of uNMN:CR and uMN:CR stability only at a single concentration within the RI. Second, urinary creatinine stability was not directly evaluated in our study, and we relied on previously published data. Third, due to the retrospective nature of the study, in six non-PHEO dogs, the hormonal secretory activity of the adrenal lesion was not determined. Finally, although the number of healthy dogs included was adequate for the establishment of laboratory RIs, the relatively small number of dogs in the disease groups, particularly those with PHEO and non-PHEO, may limit the precision and generalizability of the estimated diagnostic performance of uNMN:CR. In particular, the proposed diagnostic cut-off derived from ROC curve analysis should be interpreted with caution, as it may be influenced by sample variability and potential overlap between PHEO and non-PHEO dogs. Therefore, validation of these findings in larger and independent cohorts is warranted before widespread clinical application.

## 5. Conclusions

To the best of the authors’ knowledge, this study is the first comprehensive validation of a LC–MS/MS method for the quantification of urinary catecholamines, specifically NMN and MN, in dogs. The validated LC–MS/MS method offers a robust, sensitive, and highly selective approach for measuring these catecholamine metabolites, providing reliable RI for clinical use in canine populations. The uNMN:CR, derived using LC–MS/MS, provides an accurate and precise reflection of catecholamine metabolism. Furthermore, the ability to collect urine samples non-invasively, combined with the demonstrated stability of catecholamines in frozen specimens, makes this methodology well-suited for both routine diagnostic use and research settings. While these findings represent an important advancement, further prospective studies are required to confirm the diagnostic utility of LC–MS/MS-based urinary catecholamine measurements in dogs with various diseases, particularly pheochromocytoma and other endocrine disorders. Additionally, the impact of physiological variables such as age, sex, and diet on urinary catecholamine levels should be explored. Overall, our results support the integration of LC–MS/MS into veterinary diagnostics as a reliable, precise, and methodologically superior alternative to traditional assays for urinary catecholamines.

## Figures and Tables

**Table 1:** Accuracy of the determination of normetanephrine, in terms of the mean detected concentration. CV acceptance criteria for LLOQ: ≤20%; CV acceptance for low, medium, and high levels: ≤15%; and ME% acceptance criteria: ≤20%.

Spike Level		Intra-Assay Day 1	Intra-Assay Day 2	Intra-Assay Day 3	Inter-Assay Bias
LLOQ (1 ng/mL)	Mean (ng/mL)	1.06	1.07	1.07	1.07
	CV (%)	11.5	8.62	2.42	7.52
	Accuracy (%)	106	107	107	107
	Bias (%)	6.29	7.04	7.17	6.83
Low concentration (3 ng/mL)	Mean (ng/mL)	2.85	3.02	2.94	2.94
	CV (%)	0.97	0.68	2.33	1.33
	Accuracy (%)	95.1	100.8	98.1	98.0
	Bias (%)	−4.95	0.77	−1.89	−2.02
Medium concentration (50 ng/mL)	Mean (ng/mL)	47.2	49.5	49.1	48.6
	CV (%)	2.39	1.95	1.72	2.02
	Accuracy (%)	94.5	99.1	98.2	97.2
	Bias (%)	−5.51	−0.94	−1.83	−2.76
High concentration (100 ng/mL)	Mean (ng/mL)	99.6	99.2	94.0	97.6
	CV (%)	0.94	1.44	2.99	1.79
	Accuracy (%)	99.6	99.2	94.0	97.6
	Bias (%)	−0.44	−0.79	−6.04	−2.43
ULOQ (500 ng/mL)	Mean (ng/mL)	495	496	491	494
	CV (%)	0.57	0.61	0.77	0.65
	Accuracy (%)	99.0	99.2	98.3	98.8
	Bias (%)	−1.05	−0.77	−1.70	−1.05
ME%	Mean	85.7	111	87.2	94.9
	CV	9.39	0.99	8.00	6.13

CV (%) = coefficient of variation: (standard deviation/mean) × 100; LLOQ, lower limit of quantification; ME, matrix effect; and ULOQ, upper limit of quantification.

**Table 2:** Accuracy of the determination of metanephrine, in terms of the mean detected concentration. CV acceptance criteria for LOQ: ≤20%; CV acceptance for low, medium, and high levels: ≤15%; and ME% acceptance criteria: ≤20%.

Spike Level		Intra-Assay	Intra-Assay	Intra-Assay	Inter-Assay Bias
		Day 1	Day 2	Day 3	
LLOQ (1 ng/mL)	Mean (ng/mL)	0.97	1.03	1.02	1.01
	CV (%)	7.91	8.96	8.02	8.30
	Accuracy (%)	97.2	103	102	101
	Bias (%)	−2.78	2.71	2.38	0.77
Low concentration (3 ng/mL)	Mean (ng/mL)	2.81	2.96	2.94	2.90
	CV (%)	2.42	3.22	1.52	2.39
	Accuracy (%)	93.6	98.7	98.1	96.8
	Bias (%)	−6.38	−1.31	−1.89	−3.19
Medium concentration (50 ng/mL)	Mean (ng/mL)	48.6	49.4	48.9	49.0
	CV (%)	2.72	1.12	1.78	1.87
	Accuracy (%)	97.3	98.8	97.7	97.9
	Bias (%)	−2.7	−1.2	−2.3	−2.08
High concentration (100 ng/mL)	Mean (ng/mL)	97.6	96.8	97.6	97.3
	CV (%)	2.07	1.08	2.44	1.87
	Accuracy (%)	97.6	96.8	97.6	97.3
	Bias (%)	−2.37	−3.23	−2.44	−2.68
ULOQ (500 ng/mL)	Mean (ng/mL)	486	494	489	489
	CV (%)	2.04	1.08	2.86	2.11
	Accuracy (%)	97.2	96.8	98.0	98.0
	Bias (%)	−2.84	−3.23	−2.02	−2.00
ME%	Mean	95.5	113.9	97.2	102.2
	CV	3.07	0.67	6.29	3.35

CV (%) = coefficient of variation: (standard deviation/mean) × 100; LLOQ, lower limit of quantification; ME, matrix effect; and ULOQ, upper limit of quantification.

**Table 3:** Summary of stability testing results showing the mean, standard deviation (SD), and mean recovery percentage (%) relative to T_0_ for the quality control sample stored under various conditions (room temperature, 4 °C, −20 °C, and 40 °C) over time.

	Normetanephrine			Metanephrine		
Stability Type	Mean	SD	Mean Recovery %	Mean	SD	Mean Recovery %
T_0_	19.3	0.71		14.6	0.51	
Benchtop day 1	18.8	0.66	97.4	14.3	0.64	98.0
Benchtop day 3	19.3	0.89	99.9	15.4	0.66	105
Benchtop day 7	19.2	0.67	99.4	15.0	0.28	102
Refrigerated day 1	18.7	0.60	96.7	14.1	0.32	97.0
Refrigerated day 3	19.6	0.57	102	15.1	0.35	104
Refrigerated day 7	18.9	0.10	98	15.4	0.79	105
Frozen day 1	18.9	0.67	97.7	14.4	0.41	98.3
Frozen day 3	19.1	1.29	99.1	14.7	0.94	100
Frozen day 7	17.9	1.10	92.8	13.6	0.65	93.2
Frozen day 180	18.1	0.35	93.7	14.0	0.91	95.7
Frozen day 360	18.2	0.45	94.2	14.2	0.17	97.1
Heated day 1	20.4	0.29	105	15.6	0.38	107
Heated day 3	21.3	4.34	110	16.7	3.33	114
Heated day 7	19.2	3.45	99.6	15.5	2.96	105

**Table 4:** Normetanephrine-to-creatinine ratio in the healthy, NAI, PHEO, and non-PHEO groups, and results of their pairwise comparisons.

Group	uNMN:CR		
	Median (IQR)	Kruskal–Wallis	Pairwise Comparison
**Healthy** (*n* = 41)	34.1 (27.8–47.95)	H = 45.87 *p* < 0.001	**Healthy vs. NAI** *p* < 0.001
			**Healthy vs. PHEO** *p* < 0.001
			**Healthy vs. Non-PHEO** *p* < 0.001
**NAI** (*n* = 16)	87.7 (53.88–141.5)		**NAI vs. Non-PHEO** *p* < 0.001
**Non-PHEO** (*n* = 14)	86.15 (53.25–206.6)		**PHEO vs. Non-PHEO** *p* = 0.012
**PHEO** (*n* = 12)	414.1 (212.7–997.2)		**PHEO vs. NAI** *p* < 0.001

IQR, interquartile range (25th and 75th percentiles); NAI; dogs with non-adrenal illness; Non-PHEO, dogs with adrenal masses other than pheochromocytoma; PHEO, dogs with pheochromocytoma; and uNMN:CR, urinary normetanephrine-to-urinary creatinine ratio.

## Data Availability

The dataset is available on request from the authors due to patient and owner privacy restrictions.
